# Digitization in Skin Shade Matching for Maxillofacial Prostheses: A Systematic Review

**DOI:** 10.7759/cureus.43886

**Published:** 2023-08-21

**Authors:** Priyadarshani Pawar, Anjali G Borle, Rohit M Patil, Pradnya Patil, Vaishali M Pawar, Muskan Pachori

**Affiliations:** 1 Department of Prosthodontics, Sharad Pawar Dental College and Hospital, Wardha, Maharashtra, IND; 2 Department of Prosthodontics, Jawahar Medical Foundation (JMF) Annasaheb Chudaman Patil Memorial (ACPM) Dental College, Dhule, IND; 3 Department of Periodontology, SMBT Institute of Dental Sciences and Research, Igatpuri, IND; 4 Department of Oral Pathology, SMBT Institute of Dental Sciences and Research, Igatpuri, IND

**Keywords:** skin color, computerized methods, e-skin, maxillofacial prostheses, color matching

## Abstract

Color matching of maxillofacial prostheses for the restoration of maxillofacial defects is an important factor for esthetic results. Various methods have been introduced for the accurate and reliable color matching of prostheses with the skin color of patients. A systematic review was conducted to search the existing literature on color-matching digital techniques for maxillofacial prostheses. An electronic literature search was conducted in PubMed/Medline, Scopus, and Web of Science from January 2000 to December 2022 using Preferred Reporting Items for Systematic Reviews and Meta-Analysis (PRISMA) guidelines. Two independent reviewers conducted the search. Eight articles that fulfilled the inclusion criteria after a full-text evaluation were included in this review. Most of these studies were published in prosthodontics journals and conducted in various countries around the world. A computerized color formulation system was used in three studies; a non-contact spectroradiometer (PR 705; Photo Research Inc., Chatsworth, CA) with a Xenon arc lamp was used in two studies; a mobile phone colorimeter was used in one study; additive manufacturing of 3D facial skin with a spectrophotometer was used in one study; and a recently introduced computerized method known as e-skin (Spectromatch, Bath, UK) was used in two studies. Most of these methods were accurate in color matching, except for the additive manufacturing system, which showed less accuracy, but good repeatability. Owing to a lack of sufficient studies, no method can be labeled as the best method for color-matching maxillofacial prostheses. The latest computerized method, the e-skin, can be used to achieve better accuracy and good color matching. However, further studies are required to validate the use of e-skin for precise color matching.

## Introduction and background

Maxillofacial defects occur due to trauma, cancer, or congenital deformities and require restoration of these defects with maxillofacial prostheses. To provide good aesthetic results to the patient, precise color matching of these prostheses is very important for the patient’s skin color. Prostheses were fabricated using acrylic and silicone. The traditional method adopted for color matching was the chairside “trial and error method,” where the clinician used to add pigments to the non-polymerized silicone and kept on adding the pigment till it matched the patients’ skin color. It is a subjective assessment method with high failure rates in achieving a desirable skin color [1].

Owing to inherent problems with the subjective system, various objective systems use a digital method, where a colorimeter or spectrophotometer is used to calculate the skin color of the patients and pigment formulation is used to color the silicon [2,3]. These methods are reliable, accurate, and free from individual failures. Owing to the complexity of individuals’ skin shades, which are affected by age, gender, and ethnicity, computerized color measurement systems have been introduced [4]. One such recently introduced system is the e-skin (Spectromatch, Bath, UK) [5].

However, very few studies have compared digital methods to other methods. Only one systematic review was conducted in 2017 to review the available literature until 2017 for various color-matching methods available for maxillofacial prostheses [6]. They also concluded that there is a lack of research on this aspect. Hence, this systematic review was conducted to search the literature for various digital methods available for color matching in maxillofacial prostheses. The main question was “Which is the best digital method of color matching for maxillofacial prostheses?” The secondary questions were as follows: RQ1: Which digital methods are used for color matching? RQ2: Do results differ for different skin tones? RQ3: What other methods are used to compare results? RQ4: What are the outcomes of the methods used in this study?

## Review

Materials and methods

The Preferred Reporting Items for Systematic Reviews and Meta-Analysis (PRISMA) guidelines were followed in this review to provide clear and transparent reporting of the outcomes [7]. Ethical committee approval was not required, as this review was conducted using an online database.

Study Selection and Search Strategy

A systematic search of the English literature was conducted in PubMed/Medline, Scopus, and Web of Science for articles published between January 1, 2000, and December 31, 2022, using Medical Subject Heading Terms (MeSH terms), as shown in Table 1.

**Table 1:** Search query

Database	Query	Results
PubMed	(Color matching OR color reproduction OR color management or Skin color) AND ((Maxillofacial prostheses OR Human facial prostheses OR Maxillofacial silicone) AND (Digital methods OR colorimeter OR spectrophotometer OR e skin)	96
Scopus	All (color OR matching AND reproduction AND management AND skin) AND (Digital methods) AND (maxillofacial OR human facial AND prosthesis OR maxillofacial AND silicone)	58
Web of Science	All (Color matching OR color reproduction OR color management or Skin color) AND ((Maxillofacial prostheses OR Human facial prostheses OR Maxillofacial silicone) AND (Digital methods)	42

**Figure 1:**
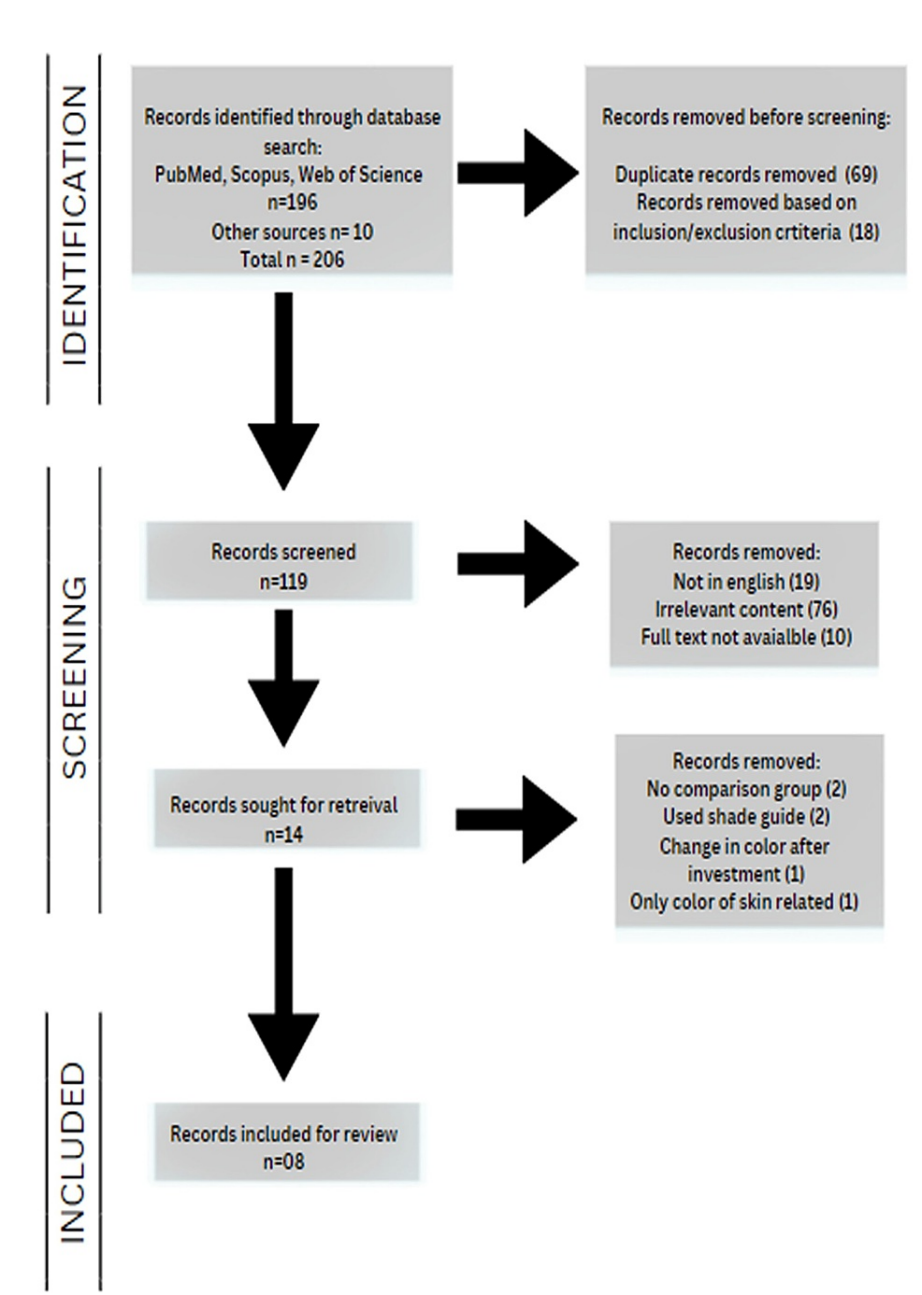
PRISMA flowchart PRISMA: Preferred Reporting Items for Systematic Reviews and Meta-Analysis

A PRISMA flow diagram of the data search is shown in Figure 1.

The first author (PP) conducted a search of the databases using MeSH terms, as mentioned in Table 1, from January 2000 to December 2022. After eliminating duplicates, two independent reviewers (AB and RP) screened the titles and abstracts of the relevant studies according to the eligibility criteria. After excluding non-relevant articles, the full text of the selected articles was further screened to remove studies that did not meet the inclusion criteria. The reasons for the exclusion of articles were recorded and reported in the review. The reference lists of the included articles were further searched for relevant articles. Any disagreements between the reviewers at each stage of the selection process were resolved through discussion until a consensus was reached.

The PICOS criteria for the search were as follows: P (population/patients) - studies involving patients with maxillofacial defects requiring maxillofacial prostheses; I (intervention), using digital methods for color matching with the skin color of the patients; Comparison of one method with at least one conventional method of assessment; O (outcome) - accuracy of digital methods in accurate color matching of maxillofacial prostheses with the skin shade of the patient.

Eligibility Criteria for the Search

Inclusion criteria:* *All retrospective and prospective cohort studies, randomized control trials, studies between January 2000 and December 2022, studies in which at least one digital method was compared with other methods of assessment, and a primary study in English were included.

Exclusion criteria:* *Case series, conference papers, theses, letters to the editor, editorials, case reports or series, animal studies, systematic reviews, meta-analyses, studies published before January 2000 and after December 2022, studies conducted in which steps other than color matching have been reported, studies where no comparison has been performed with other methods of assessment, studies conducted on postmortem data or non-humans, and studies published in any language other than English were excluded.

Data Extraction

Data were extracted according to the PICOS criteria. Information on author name, year and type of publication, journal or source name, type of method used, purpose of the study, details of demographic data such as number of subjects, ethnicity, type of other methods used for assessment, and outcome of the intervention were extracted from the studies.

Results

Meta-analysis could not be performed in the present systematic review due to the presence of high heterogeneity in the studies, regarding their outcome measurement, comparison between the groups, study design, study population, and target parameters. The initial search of the databases resulted in 196 articles; an additional 10 records were identified through additional sources. There was perfect agreement between the two reviewers (kappa value = 0.95) in the initial screening of titles and abstracts. Any disagreements were resolved by a third reviewer. Thirteen studies were selected after going through full-text articles. Seven studies were excluded because there were no comparisons with other methods in two studies [8,9], one study evaluated only skin color and not prostheses or silicone samples [10], and only shade guides were used in two studies [11,12], and one study evaluated the change in color after investment [13]. Finally, eight studies were included [2-5,14-17], as shown in the PRISMA flow diagram (Figure 1).

Data Overview of the Included Studies

All included studies were prospective. Most of these studies were published in prosthodontics journals, except for three studies that were published in non-prosthodontic journals [14,15,17]. The results revealed that two studies were conducted in Turkey [5,16], one in Ireland [4], one in Manchester [17], one in Iraq [15], one in Columbus [14], and two in Canada [2,3]. A computerized color formulation system was used in two studies to fabricate pigmented silicone samples and compare them with spectrophotometer color-matched samples [2,3]. Three studies used an e-skin spectrocolorometric computerized color formulation system with a mobile phone colorimetric method [4], target skin color based on a spectrophotometric method [16], and visual examination of patients’ skin shade by three observers [5]. One study used an additive manufacturing process for pigmented soft tissue facial prostheses and compared it with two skin shades using spectrophotometric methods [17]. One study compared two contact methods, colorimeter and spectrophotometer, with non-contact methods, PR 705 spectroradiometer, and fiber-optic light cable connected to a Xenon Arc lamp [14]. Four studies were conducted on subjects of different ethnicities: African-Canadian [2,3], Caucasian-Chinese [17], and Caucasian-Chinese-Asian-African-Caribbean [15]. Most digital methods used for color matching showed acceptable accuracy, except for the additive manufacturing method, which showed poor accuracy but good reproducibility [17], as shown in Table 2.

**Table 2:** Details of the included studies

Author	Year	Purpose	Type of study, Participants, Ethnicity	Intervention	Outcome
Coward TJ et al. [2]	2008	The effectiveness of the digital method for the color matching of silicone elastomer with skin color	Prospective study on 19 African-Canadian	Spectrophotometry and a computerized color formulation system	Delta E values of 1.49±2.2, a good match with skin color.
Hu X et al. [14]	2010	Comparison of contact and non-contact methods for color matching	Prospective study on 24 silicone specimens matched with a mini ColorChecker chart	Contact methods were colorimeter and spectrophotometer, Non-contact were PR 705 spectroradiometer, Fibre optic Light cable connected to Xenon Arc lamp.	Non-contact methods performed better.
Seelaus R et al. [3]	2011	Comparison of the computerized method with clinical judgment for color matching	Prospective study in African-Canadian	Silicone samples matched with the skin color based on spectrophotometric measurements and computerized color formulation	Low delta E values and good reflectance values for skin color and silicone samples. Increased pigmentation, High E values
Xiao K et al. [15]	2013	To develop and test the accuracy of color reproduction systems in advanced manufacturing technology	Prospective study on 14 skin colors (4 Caucasian, 2 Chinese, 2 Asian, 4 African, and 2 Caribbean)	3D printing system matched with skin colors based on spectrophotometric measurements	Acceptable Delta E values, good accuracy but less repeatability, particularly for extreme dark or light shades.
Nemli SK et al. [16]	2018	Accuracy of color and translucency matching of the e-skin system across different skin colors	Prospective study on 28 skin colors	The e-skin system compared with spectrophotometer target skin shades	For translucency (TP), the CIELAB Delta E value was 0.663, with good reliability and accuracy. Mean TP values of e-skin are higher (8.8) than target colors (7.6).
Sohaib A et al. [17]	2018	Color quality was assessed throughout the additive manufacturing process,	Prospective study on 2 skin types, Caucasian and Chinese	Additive manufacturing of 3D facial skin compared with 2 skin colors based on spectrophotometric measurements	CIEDE 2000 values were 3.6±1.18, larger than acceptable values, not good accuracy but good reproducibility (0.34 to 0.89)
Mulcare DC et al. [4]	2020	Compare the accuracy of mobile phone colorimeter (MPC) with e-skin	Prospective study on 10 pigmented silicone samples with e-skin	e-skin system pigmented 10 silicone samples were compared with MPC	L*A*B* and Delta E values were good and within acceptable limits.
Kurt M et al. [5]	2021	To determine the color match of silicone replicas fabricated with e-skin	Prospective study on 30 participants (15 of dark skin and 15 of light skin)	e-skin silicone samples were compared with patients’ skin color by 3 observers	Non-significant difference. Mean L*A*B* values of light skin were higher for e-skin samples. Excellent agreement between observers for patient skin color and e-skin samples.

Discussion

Facial prostheses serve as replacements for lost facial features that cannot be reconstructed using a patient's own tissues. For such prostheses to be deemed successful, it is imperative that they be realistic. The color-matching process, which involves matching silicone to the skin, is of utmost importance in ensuring the success of the prosthesis and the overall rehabilitation of the patient. However, owing to the effects of metamerism, achieving an exact color match poses a considerable challenge [6].

Various methods have been introduced to pigment non-polymerized silicone for accurate matching with the skin tone of patients such as the spraying technique [18], color blending with external color tinting [19], tattooing [20], milling with extrinsic colors [21], commercially available cosmetic products for shade matching [22], and shade guides [23]. Due to variability in the skin tone of the patients, based on age, sex, ethnicity, and different translucency of the skin, none of the methods could produce accurate results for color matching. Therefore, digital methods have been developed and found to be accurate within acceptable limits to match the shade of pigmented silicone prostheses with the patient’s skin color [2-5,14-17].

No systematic or literature review has been conducted recently to assess the accuracy of the various digital methods used for color-matching of maxillofacial prostheses. Only one systematic review was conducted on this topic in 2017, which included studies until 2015 and found very few studies conducted on this topic [6].

Skin color is determined by a combination of five pigments, namely, melanin, melanoid, reduced hemoglobin, oxyhemoglobin, and carotene, which are present in different layers of the skin. The unique absorptive capacity of melanin determines the value, hue, and saturation of skin color [24]. Computerized methods have been employed to formulate different color formulations to mimic skin color [2,3,5,7,8]. Studies have shown that the accuracy of these systems varies with different skin tones present in patients of different ethnic origins [2,3,17]. This might be due to the difference in melanin present in the skin, which affects the color of the hue. Coward et al. observed that the process of color matching is more arduous when performed on individuals with dark skin tones [2]. Similar findings were reported by Xiao et al., who observed reduced accuracy of color reproduction using a 3D color printing system for extremely dark and bright skin colors [15]. They concluded that as the amount of melanin in the skin increased, the Delta E values for color matching increased, showing less accuracy. In direct opposition, the findings of Nemli et al. demonstrated that CIELAB Delta-E values are reduced in instances where darker hues are utilized compared to their lighter counterparts [16]. The authors suggested that this may be attributed to the heightened density of melanin pigment present within darker skin tones, which could lead to greater applicability of brown or similar pigments to tested color-matching systems for replication purposes.

The dissimilarities in the spectral properties exhibited by human skin and silicone, as well as the non-uniformity of the former, could potentially explain the phenomenon of color mismatch observed between silicone replicas and skin. In color formulation procedures, the employment of Delta E values serves as a benchmark for ascertaining the degree of similarity between two given colors. Troppmann et al. have propounded that a Delta E value of 1.75 or lower can be deemed as an arbitrary tolerance limit for the clinical application of color formulation [25]. However, there is no formally established tolerance limit for a clinically acceptable Delta E value for silicone elastomers in the existing literature. Thus, further investigations are warranted to determine the clinically acceptable Delta E value for facial prosthetic restorations in patients of different ethnicities. Most studies conducted on the use of various digital methods for color matching had acceptable Delta E values, except for one study that used additive manufacturing processing for the fabrication of 3D facial skin prostheses [17]. In addition, Delta E and L*A*B* values were also matched between silicone samples and the patient’s skin color (L indicates lightness or brightness, A indicates redness or greenness in the sample, and B indicates yellowness or blueness in the sample). Both the CIELab and CIEDE2000 color-difference formulas have been utilized for dental color research. Nevertheless, the CIEDE2000 color-difference formula has been deemed more trustworthy in terms of providing accurate indications of human perceptibility and acceptability of discrepancies in tooth color. Consequently, it has been strongly recommended for implementation in the context of clinical instrumental color analysis [17].

The human skin is a multifunctional and partially translucent biomaterial with intricate optical properties. Because of the deep penetration of light through the skin, it is challenging to ascertain and compute its translucency, as with other translucent materials of a specific thickness. Therefore, the measurement of translucency in human skin remains an ongoing challenge for researchers. In the realm of human skin, the measuring spot of a color-measuring device is unable to reflect light within the detection area, leading to subsurface scattering and absorption during measurement, commonly known as "edge loss" [26]. Given the context of computerized color matching of maxillofacial silicone, there may be certain apprehensions regarding the optical behavior of the silicone and skin. Nevertheless, it is important to note that edge-loss errors have also been observed in translucent pigmented elastomers, indicating that this issue is not exclusive to silicone and the skin. As such, it is imperative that researchers and practitioners alike exercise caution and vigilance in their approach toward color matching and measurement in order to minimize the impact of edge loss and other related factors. In a study by Hu et al., who compared two contact methods with non-contact methods for color matching, it was observed that the contact methods had more edge loss than the non-contact methods [14].

The accurate color reproduction of a silicone maxillofacial prosthesis is a complex process that involves several factors, including the proper mixing of pigments in appropriate quantities. The silicone mixing technique is a significant factor that affects the color reproducibility of the final product. The presence of air voids and pores within silicone can scatter the reflected light, which ultimately influences the overall color of the specimen, making it a crucial consideration in the color-matching process [27]. To attain a maxillofacial prosthesis that blends seamlessly with the surrounding skin, it is essential to consider variations in skin color across the defect and face by conducting meticulous color measurements and mixing.

Recently, the field of maxillofacial prostheses has witnessed the introduction of a new color-matching system, namely, e-skin, developed by Spectromatch Ltd. This technology involves the use of a portable contact spectrocolorimeter that records color measurements with respect to the standard illuminant D65 and 10-degree observer angle [16]. The introduction of this system has great potential for facilitating accurate matching of skin tones for use in maxillofacial prosthetics. After measuring the skin area of interest, the device provides a color code that is used to procure the color formulation via an online calculator (https://www.spectromatch.com/calculator/). This process is imperative for acquiring precise color information required for tasks such as cosmetics, textiles, and printing. The use of such technology not only facilitates quality control but also enhances the efficiency of color-matching processes. The e-skin system has been used in limited studies and provides skin replicas that match the skin color within clinically acceptable thresholds [5,16].

Recently, the application of deep learning has become an everyday phenomenon because of its superiority in prediction and classification tasks. Inspired by the human brain, artificial neural networks (ANN) have made substantial progress in recent years. The primary impetus behind this progress is the assimilation of nonlinear dependencies associated with input data by employing a combination of linear systems (referred to as convolutions in layers) and nonlinear differentiable activation functions. Notably, research in this field remains limited and Deep Learning (DL) models have demonstrated good accuracy. However, no comparisons have been made using manual methods [28].

Matching the color of the maxillofacial prosthesis with the human skin tone within the threshold of acceptability is often a significant challenge for a maxillofacial prosthodontist. Recording the exact human skin tone and replicating it in the prosthesis is hard. Many techniques have been documented in the literature to achieve color matching in the maxillofacial prosthesis. Most of the time, it has been done with a trial and error method, which compromises on the exact color matching with the patients' skin color. This systematic review presented herein failed to provide any evidence regarding the best technique for achieving perfect color matching in the fabrication of facial prostheses. This was primarily due to the heterogeneity of the included studies as well as the limited number of studies conducted on each method. Nevertheless, it is believed that more recent techniques, such as e-skin, computerized formulations, and mobile phone colorimetry, have significantly improved the efficiency of color matching. Therefore, further studies are necessary to assess the accuracy of these color-matching methods.

## Conclusions

Color matching is a critical stage in the production of maxillofacial prostheses. There are several approaches to matching the color of the facial skin in maxillofacial prosthetics. The introduction of new techniques has made the coloration process more precise and time-consuming. This systematic review showed that the trial-and-error method is the most commonly used technique in clinical practice for the color matching of facial prostheses. Despite limited data on the color matching of facial prostheses, there is no current evidence to suggest that one technique is superior to the other. Nevertheless, careful consideration should be given to selecting the most appropriate color-matching technique based on the patient's individual needs and the clinician's expertise. Ultimately, the goal is to achieve a natural and esthetically pleasing appearance.
